# What Constitutes Traditional and Modern Eating? The Case of Japan

**DOI:** 10.3390/nu10020118

**Published:** 2018-01-25

**Authors:** Gudrun Sproesser, Sumio Imada, Isato Furumitsu, Paul Rozin, Matthew B. Ruby, Naomi Arbit, Claude Fischler, Harald T. Schupp, Britta Renner

**Affiliations:** 1 Department of Psychology, University of Konstanz, P.O. Box 47, 78457 Konstanz, Germany; britta.renner@uni-konstanz.de; 2 Department of Psychology, Hiroshima Shudo University, 1-1-1 Ozukahigashi, Asaminami, Hiroshima 731-3195, Japan; imada@shudo-u.ac.jp (S.I.); ifurumit@shudo-u.ac.jp (I.F.); 3 Department of Psychology, University of Pennsylvania, 3720 Walnut St./Solomon Labs Building, Philadelphia, PA 19104, USA; rozin@psych.upenn.edu; 4 Department of Psychology and Counselling, La Trobe University, P.O. Box 821, 133 McKoy Street, Wodonga, VIC 3690, Australia; m.ruby@latrobe.edu.au; 5 Teachers College, Columbia University, 525 West 120th Street, New York, NY 10027, USA; naomi.arbit@gmail.com; 6 CNRS, Ecole des Hautes Etudes en Sciences Sociales, 75006 Paris, France; fischler@ehess.fr; 7 Department of Psychology, University of Konstanz, P.O. Box 36, 78457 Konstanz, Germany; harald.schupp@uni-konstanz.de

**Keywords:** traditional eating, modern eating, Japan, multi-faceted, dimensions, compilation, systematization

## Abstract

Traditional Japanese dietary culture might be a factor contributing to the high life expectancy in Japan. As little is known about what constitutes traditional and modern eating in Japan, the aims of the current study were to (1) comprehensively compile and systematize the various facets of traditional and modern eating; and (2) investigate whether these facets also apply to traditional and modern eating in Japan. In Study 1, an extensive international literature review was performed. Forty-five facets of traditional and modern eating were compiled and systematized into the dimensions of what and how people eat, and into eleven separate subdimensions. In Study 2, 340 adults from Japan answered a questionnaire. Results showed that traditional and modern eating in Japan is reflected in both what and how people eat. Within these two dimensions, ten subdimensions were found: the ingredients, processing, temporal origin, spatial origin, and variety of consumed foods, as well as temporal, spatial, and social aspects, appreciation, and concerns when eating. This study provides a broad compilation of facets of traditional and modern eating in Japan. Future research should investigate how these facets are related to life expectancy and health.

## 1. Introduction

In 2016, the World Health Organization reported that life expectancy in Japan was approximately 84 years, the highest life expectancy worldwide [1]. The traditional Japanese dietary culture has been proposed as one factor contributing to this high life expectancy [2]. Also, the traditional Japanese cuisine (‘Washoku’) was designated as Intangible Cultural Heritage by the UNESCO in 2013 [3] and is of global interest. At the same time, the worldwide transition towards modern eating behavior is also occurring in Japan [4]. However, what exactly constitutes traditional and modern eating behaviors in Japan?

When trying to answer this question, previous research has mostly focused on what people eat—more precisely, on the ingredients of Japanese meals [2,4,5,6,7,8]. For instance, traditional eating behavior in Japan has been characterized by high consumption of cereals (rice), soybean products, vegetables, fish and salt, as well as by a low to moderate energy content of meals. In contrast, modern eating behavior has been characterized by high consumption of fat, (refined) sugar, meat, dairy products, and eggs [2,4,5,6,7,8,9]. Besides these meal ingredients, food processing and dietary variety have also been put forth as aspects of traditional and modern Japanese eating behavior. Specifically, industrially processed foods and a diverse diet are part of modern eating behavior in Japan, whereas traditional eating behavior encompasses the consumption of fresh foods and few food choices [4,5].

A review of international literature shows, however, that traditional and modern eating behaviors also encompass how people eat. First, temporal aspects of eating behavior have been mentioned. For instance, Fjellström [10] stated that traditional eating behavior is characterized by eating at predetermined mealtimes. Snacking, in contrast, seems to be part of modern eating behavior [11,12]. Second, spatial aspects have been brought up. Specifically, eating at home is considered part of traditional eating, whereas eating out of home and on the run are considered part of modern eating [11,13,14,15,16]. Third, social aspects have been named. More precisely, traditional eating behavior is characterized by eating together [11,13,17] and according to collective rules with others deciding what to eat [18]. In contrast, modern eating behavior is characterized by eating alone [19] and eating according to individual preferences [18]. Fourth, the appreciation of food has been mentioned, with focus on the meal and others at the table being a marker of traditional eating behavior, and focus on something else while eating, such as using the mobile phone or reading the newspaper, as a marker of modern eating behavior [13]. Lastly, traditional and modern eating seem to differ in the concerns that people have. Fischler [18] states that traditionally, people were concerned about the availability of food, whereas in modern times, people are concerned about the quality of food and which foods to choose.

Taken together, traditional and modern eating behaviors are highly complex. First, previous research suggests multiple facets of traditional and modern eating behavior, such as the consumption of vegetables or sugar. Second, these facets can be categorized into different subdimensions of traditional and modern eating, such as the ingredients of meals. Third, these subdimensions can again be subsumed into different dimensions, such as what or how people eat. Presently, no one has comprehensively compiled these various facets and systematized them into subdimensions and dimensions of traditional and modern eating behaviors. Also, it remains unknown whether the facets, subdimensions, and dimensions mentioned in the international literature capture traditional and modern eating behaviors in Japan. Hence, the aim of Study 1 was to provide a broad compilation and systematization of facets, subdimensions, and dimensions of traditional and modern eating behavior. The aim of Study 2 was to investigate whether these facets, subdimensions and dimensions also apply to traditional and modern eating in Japan, by assessing the perceptions of Japanese laypeople. Importantly, men and women might differ in their judgement of how traditional or modern certain aspects are, because women are mostly responsible for food purchasing and preparation [5,20]. Also, younger and older people might differ. Hence, we aimed to find aspects that are seen as traditional or modern in all of these groups.

## 2. Study 1

To provide a broad compilation and systematization of facets, subdimensions, and dimensions of traditional and modern eating behavior, the authors performed an extensive international literature review and held a series of discussions. The literature review was performed in major databases (e.g., Web of Science, Google Scholar). Several combinations of the terms traditional, modern, food, eating, and nutrition transition were used. Also, references of relevant articles were screened and books were reviewed. No limits were established regarding the year of publication, but only English literature was included in the review. The literature review and discussions revealed 45 facets of traditional and modern eating behavior (see Table 1). Using the Constant Comparative Method [21], two researchers sorted these 45 facets into categories, by grouping similar facets. This procedure resulted in two dimensions: (1) what people eat; and (2) how people eat. Subdimensions in the dimension ‘what people eat’ were Ingredients, Processing, Preparation, Temporal Origin, Spatial Origin, and Variety. Subdimensions in the dimension of ‘how people eat’ were Temporal Aspects, Spatial Aspects, Social Aspects, Appreciation, and Concerns.

In sum, Study 1 provided a broad compilation and systematization of facets, subdimensions, and dimensions of traditional and modern eating behaviors. In Study 2, we investigated whether these facets, subdimensions, and dimensions also apply to traditional and modern eating in Japan, by assessing the perceptions of Japanese laypeople.

## 3. Study 2

### 3.1. Materials and Methods

#### 3.1.1. Participants and Procedure

In total, 340 participants took part in this study. The younger group of participants (*n* = 140) had a mean age of 19 years (SD = 1.3; range: 18–21 years) and included 60% women (*n* = 84). The older group of participants (*n* = 200) had a mean age of 63 years (SD = 7.7; range: 50–86 years) and included 50% women (*n* = 100). The younger group of participants were all undergraduate students, enrolled in psychology classes at Hiroshima Shudo University. The questionnaire was handed out to each individual who consented to take part in this study. The older group had been living in Japan for more than 18 years. They were recruited via a company that specialized in academic research, with a panel of more than 300,000 older registrants, who completed the questionnaire online. The ethics board of the Hiroshima Shudo University approved the study protocol (ethic approval code: 2017PSY01). The study conforms to the Declaration of Helsinki. Older participants consented to participate in this study by ticking a respective box at the beginning of the survey after being fully informed about the study. Younger participants gave written informed consent before completing the questionnaire.

#### 3.1.2. Measures

**Figure 1:**
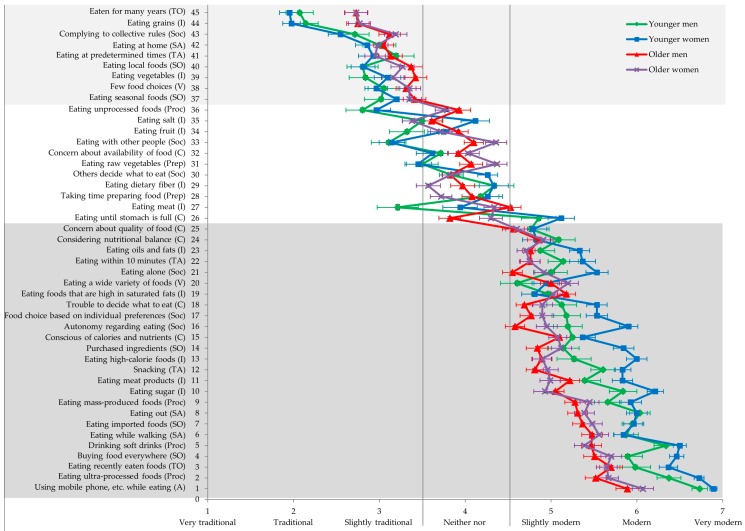
Mean rating of the 45 facets by age and gender. Facets are sorted by their overall mean. Error bars indicate standard errors of the mean. Related subdimensions (abbreviations, see Table 1) are written in parentheses after abbreviated items.

For each facet collated in Study 1, an item was generated with a 7-point rating scale, from 1 ‘very traditional’ to 7 ‘very modern’. Abbreviated items are displayed in Figure 1, and full items are listed in the Appendix A. Participants were instructed to rate to what degree each item represents traditional or modern eating behavior in Japan. English items were translated into Japanese, using the back-translation method [30]. Differences between the original and back-translation were solved by discussion and amendment of the Japanese wording if possible. If amending the Japanese wording was not possible, the original English wording was amended.

#### 3.1.3. Analytical Procedure

Statistical analyses were conducted using IBM SPSS (Version 24 for Windows). Facets were classified as traditional eating behavior if they had a mean below 3.5, with a value of 1 meaning ‘very traditional’, 2 ‘traditional’, and 3 ‘slightly traditional’. To get reliable and consistent results, facets were only classified as traditional if they had a mean below 3.5 in all four groups; that is younger and older men, as well as younger and older women. Similarly, facets were classified as modern eating behavior if they had a mean of 4.5 or higher, with a value of 5 meaning ‘slightly modern’, 6 ‘modern’, and 7 ‘very modern’. Again, we required a mean of 4.5 or higher in all four groups. If a facet’s mean was between 3.5 and 4.499, in at least one of the four groups, the facet was classified as neither traditional, nor modern, as a value of 4 indicated ‘neither nor’.

### 3.2. Results

Means and standard errors of the four groups (younger and older men, younger and older women) are displayed in Figure 1. Means and standard errors for the overall sample as well as for the four groups are also presented in the Appendix A.

#### What Constitutes Traditional and Modern Eating Behavior in Japan?

In all four groups, nine facets, representing seven subdimensions and both dimensions, had a mean below 3.5, and were thus classified as part of traditional eating behavior in Japan (see Figure 1). Twenty-five facets, representing 10 subdimensions and both dimensions, had a mean of 4.5 or higher in all groups and were thus classified as modern eating behavior in Japan. Eleven facets had means between 3.5 and 4.499 in at least one of the four groups, and were thus classified as neither traditional nor modern.

Concerning the dimension of ‘what people eat’, within the subdimension of Ingredients, two facets were rated as traditional (eating grains and vegetables) and five facets were rated as modern (consumption of meat products, oils and fats, saturated fats, high-caloric foods, and sugar), whereas four facets were rated as neither traditional nor modern in at least one of the four groups (consumption of salt, fruit, fiber, and meat). For the subdimension of Processing, three facets were rated as modern (consumption of ultra-processed foods, mass-produced foods, and soft drinks), whereas the facet of traditional eating (consumption of unprocessed foods) was rated as neither traditional nor modern in at least one of the four groups. For the subdimension of Preparation, neither of the facets (eating raw vegetables; taking time preparing foods) was rated as traditional or modern, suggesting that this subdimension does not reflect traditional and modern eating behaviors in Japan. For the subdimension of Temporal Origin, eating foods that were eaten in the area for many years was rated as traditional, whereas eating foods that have only recently been eaten was rated as modern. Similarly, all facets within the subdimensions of Spatial Origin and Variety were in line with previous research. Specifically, the consumption of locally-produced and seasonal foods (subdimension Spatial Origin) as well as few food choices (subdimension Variety) were rated as traditional, whereas purchased ingredients, imported foods, buying food everywhere (subdimension Spatial Origin), and eating a wide variety of foods (subdimension Variety) were rated as modern, in all four groups.

Concerning the dimension of ‘how people eat’, all facets within the subdimensions of Temporal and Spatial Aspects were in line with previous research. More precisely, eating at predetermined mealtimes (subdimension Temporal Aspects) and eating at home (subdimension Spatial Aspects) were rated as traditional, whereas eating within ten minutes, snacking (subdimension Temporal Aspects), eating out, and eating while walking (subdimension Spatial Aspects) were rated as modern. For the subdimension of Social Aspects, one facet of traditional eating (collective rules) and all three facets of modern eating (eating alone, food choice based on individual preferences, autonomy regarding eating) were in line with previous research. Two facets of traditional eating were rated as neither traditional nor modern (eating with others, others decide what to eat). For the subdimensions of Appreciation and Concerns, all facets of modern eating were in line with previous research (Appreciation: using mobile phone while eating. Concerns: quality of food, nutritional balance, trouble deciding what to eat, conscious of calories and nutrients). However, the two facets of traditional eating within the subdimension of Concerns were rated as neither traditional, nor modern, in at least one of the four groups (availability of foods, eating until full).

It is important to note that there were both gender and age differences in the rating of certain facets (see Figure 1 and Table A2 for MANOVA results). For instance, younger participants rated ‘eating grains’ as ‘traditional’ (mean of 2), whereas older participants rated this facet as ‘slightly traditional’ (mean of 3). However, although younger and older participants disagreed on how traditional ‘eating grains’ was, they still agreed that ‘eating grains’ is traditional rather than modern.

## 4. Discussion

Study 1 provided a broad compilation of 45 facets of traditional and modern eating behaviors from an extensive international literature review and authors’ discussions. These were subsumed under two dimensions and eleven subdimensions. The dimension of ‘what people eat’ comprised the subdimensions of Ingredients, Processing, Preparation, Temporal Origin, Spatial Origin, and Variety. The dimension of ‘how people eat’ comprised the subdimensions of Temporal Aspects, Spatial Aspects, Social Aspects, Appreciation, and Concerns. Study 2 investigated which of these facets, subdimensions, and dimensions constitute traditional and modern eating in Japan by querying younger and older men and women. Results showed that ten of the eleven subdimensions were considered part of traditional and modern eating behaviors in Japan, encompassing both dimensions of what and how people eat. In total, nine facets were found to represent traditional eating behavior in Japan, whereas 25 facets were found to represent modern eating behavior. To the best of the authors’ knowledge, Study 1 is the first study to provide a broad compilation and systematization of the various facets of traditional and modern eating behavior. Given the multi-faceted structure of traditional and modern eating behaviors, future research needs to shed light on which characteristics are related to life expectancy and health.

Importantly, both dimensions, and 10 out of 11 subdimensions of traditional and modern eating behavior were considered valid in Japan. Only one subdimension (Preparation), and 11 out of 45 items, were regarded as neither traditional nor modern. Within the subdimension of Ingredients, four out of 11 facets were classified as neither traditional nor modern in the present study (eating salt, fruit, fiber, and meat), although previous research suggests that salt consumption is traditional in Japan [4,5] and international research classifies meat consumption as modern [4,6,7] and fruit and fiber consumption as traditional [14,22,23,24]. Reasons for this might be that (1) international research suggests that high salt consumption is characteristic of modern eating behavior [15,26], yet salt consumption in Japan might not differ between traditional and modern eating, but instead, might be a marker of both; (2) items might have been formulated suboptimally—e.g., ‘fiber’ being hard for lay people to translate into specific foods; and (3) eating a little bit of meat might be traditional in Japan, but eating a lot of meat might be modern [4]. How much people eat of certain foods might even be another facet of traditional and modern eating, broadening the compilation presented in Study 1.

Within the subdimension of Processing, one out of four facets was rated as neither traditional nor modern (eating unprocessed foods), although the consumption of fresh foods has been described as part of traditional eating in Japan [5]. This contradiction might have resulted from not emphasizing in the item that the food should be industrially unprocessed, but can still be manually cooked or processed. Regarding the subdimension of Preparation, both items in this subdimension were generated through authors’ discussions, but not regarded as part of traditional and modern eating in Japan. Interestingly, Prescott et al. [31] found that convenience aspects when choosing foods were least important for people in Japan, compared to people in New Zealand, Taiwan, and Malaysia. Future qualitative research is needed to examine whether the subdimension of Preparation indeed does not play any role in traditional and modern eating behaviors in Japan. Finally, within the subdimension of Social Aspects, ‘eating with others’ and ‘others decide what to eat’ were regarded as neither traditional nor modern, although international research classified these facets as traditional [11,13,18]. A reason for this might be that ‘others’ have to be clarified, as eating with family members might be traditional in Japan, whereas eating with colleagues might be modern.

Another point for discussion is that more subdimensions and facets were found for modern eating in Japan than for traditional eating in Japan. Specifically, nine facets, representing seven subdimensions, were regarded as traditional in Japan, whereas 25 facets, representing 10 subdimensions, were regarded as modern. This might have resulted from the fact that mainly facets from international literature were included. Whereas there seem to be marked country differences in what constitutes traditional eating behavior, modern eating behavior is more homogenous across countries. Specifically, Bach-Faig et al. [32] (p. 2274) speak about “the homogenisation of food behaviours in the modern era”. Hawkes [33] (p. 13) takes this further, stating that “globalization is often viewed as coca-colonization or McDonaldization—a homogeneous process with homogeneous outcomes”. To uncover more facets and subdimensions of traditional eating in Japan, future studies might need to take qualitative, ethnographic approaches and ask open-ended questions about traditional eating behavior in Japan. Still, it is important to note that both dimensions—what and how people eat—as well as seven out of 11 subdimensions, were represented to apply to traditional eating in Japan.

The current study took a conservative approach, by classifying only those facets as traditional or modern that were rated to be so by younger and older men and women. Still, these groups differed in their judgments of how traditional or modern certain facets were. Interestingly, age differences were larger than gender differences (see Figure 1 and Table A1 and Table A2). This might be explained by the fact that one definition of traditional eating behavior is behavior as it was before World War II [29]. Older age makes people more familiar with this behavior, while gender is not discriminant in this respect. Younger people, in contrast, are more likely to adapt new things [34] and should thus be more familiar with modern behavior. Another explanation for the observed age differences might be that younger people have been found to use more extreme response options than older people in questionnaire research [35]. Interestingly, gender differences in ratings were larger for the modern items than for the traditional items, especially among the younger participants. Specifically, younger women rated most modern items higher than did younger men. This might be due to (especially younger) women being more preoccupied with food than men [36]. In sum, although the groups of participants disagreed on the extent of how traditional or modern certain facets were, they agreed that nine facets, representing seven subdimensions, were traditional rather than modern, and that 25 facets, representing 10 subdimensions, were modern rather than traditional.

### Limitations

Some limitations of the present study need to be considered. First, our sample might not be representative, in terms of socioeconomic status (SES) or living area. SES and whether people grew up in a rural or urban area might impact ratings of what constitutes traditional and modern eating behavior, and this needs to be addressed in future research. Second, as stated above, we chose a quantitative approach based on previous research, rather than an open-ended qualitative approach, to investigate what constitutes traditional and modern eating behaviors. The latter should be taken up in future research to reach a more nuanced understanding of traditional and modern eating behavior in Japan. For instance, green tea consumption might be a further facet of traditional eating in Japan.

## 5. Conclusions and Implications for Future Research

The present research showed that traditional and modern eating behavior in Japan goes beyond what people eat, encompassing more than just ingredients (e.g., vegetables), the processing of foods, and dietary diversity. Over and above this ‘what’-dimension, the present work showed the importance of ‘how’ people eat, including five subdimensions. Moreover, two additional subdimensions within the ‘what’-dimension—the temporal and spatial origin of foods—emerged as important aspects of traditional and modern eating behaviors in Japan. Given that traditional and modern eating behaviors in other countries are similarly multi-faceted, future research on the transition towards modern eating in different countries might profit from broadening its focus on ingredients to also include how people eat.

Moreover, future research needs to shed light on which characteristics are related to life expectancy and health, in order to uncover how the traditional Japanese dietary culture contributes to Japan’s high life expectancy. Previous international research seems to indicate that certain characteristics are related to health risk parameters and adverse health outcomes—a verification is needed in the case of Japan. For instance, consumption of ‘modern’ ingredients, such as saturated fats or sugar, consumption of ’ultra-processed foods’, eating away from home, and eating alone have been discussed to be related to obesity, hypertension, diabetes, or mortality [14,15,19,37]. In addition, it makes sense that the facets that have been shown to be traditional in Japan, for instance, the consumption of locally-produced and seasonal foods, respecting natural resources, or the compliance to collective rules promote a healthy weight and, thus, prevent diseases. However, empirical data are needed to test the relationship between these facets and health-related parameters.

## Figures and Tables

**Table 1:** Facets, subdimensions, and dimensions of traditional and modern eating behavior.

Subdimension	Facets of Traditional Eating Behavior	Facets of Modern Eating Behavior
**Dimension ‘what people eat’**		
Ingredients (I)	Grains [4,22] Vegetables [4,5,15,23] Salt [4,5] Fruit [23] Fiber [14,22,23,24]	Meat [4,6,7] Meat products [4] Oils and fats [4,14,15,22,23,24,25,26,27] Saturated fats [4,14,22] High calorie content [23,26] Sugar [4,5]
Processing (Proc)	Unprocessed foods [5,28]	Ultra-processed foods [4,14,26] Mass-produced foods [29] Soft drinks [23]
Preparation (Prep)	Raw vegetables (authors) Taking time preparing food (authors)	
Temporal Origin (TO)	Foods that were eaten in area for many years [29]	Foods that have only recently been eaten [29]
Spatial Origin (SO)	Locally produced food [29] Seasonal foods (authors)	Purchased ingredients (authors) Imported foods [15,29] Buying food everywhere (authors)
Variety (V)	Few food choices [4]	Eating a wide variety of foods [4]
**Dimension ‘how people eat’**		
Temporal Aspects (TA)	Predetermined mealtimes [10]	Eating within 10 min (authors) Snacking [11,12]
Spatial Aspects (SA)	Eating at home [13,14,15]	Eating out [13,16] Eating while walking [11,13]
Social Aspects (Soc)	Collective rules [18] Eating with others [11,13] Others decide what to eat [18]	Eating alone [19] Food choice based on individual preferences [18] Autonomy regarding eating [18]
Appreciation (A)		Using mobile phone while eating [13]
Concerns (C)	Availability of food [18] Eating until full (authors)	Quality of food [18] Nutritional balance (authors) Trouble deciding what to eat [18] Conscious of calories and nutrients (authors)

## Appendix A

**Table A1:** Items, means (*M*) and standard errors (*SE*) for the total sample and the four groups.

	Overall (*n* = 340)		Younger Men (*n* = 56)		Younger Women (*n* = 84)		Older Men (*n* = 100)		Older Women (*n* = 100)	
Item	*M*	*SE*	*M*	*SE*	*M*	*SE*	*M*	*SE*	*M*	*SE*
Eating food that has been eaten in this area for many years	2.43	0.07	2.07	0.16	1.95	0.11	2.73	0.14	2.73	0.13
Eating grains	2.46	0.07	2.14	0.15	1.98	0.10	2.75	0.13	2.77	0.13
Strictly complying to collective rules (e.g., of the family, community, society)	2.93	0.07	2.71	0.17	2.55	0.14	3.12	0.12	3.19	0.13
Eating at home	2.97	0.07	3.00	0.19	2.86	0.13	3.05	0.12	2.98	0.12
Eating breakfast, lunch, and dinner at pre-determined times	3.04	0.08	3.20	0.21	2.93	0.17	3.13	0.14	2.95	0.12
Eating locally produced food	3.11	0.07	2.80	0.18	2.81	0.14	3.37	0.14	3.27	0.13
Eating vegetables	3.17	0.08	2.84	0.19	3.10	0.15	3.42	0.13	3.16	0.14
Having few food choices	3.19	0.07	3.05	0.18	2.96	0.13	3.31	0.12	3.35	0.13
Eating vegetables and fruit of the season	3.28	0.08	3.02	0.19	3.20	0.16	3.41	0.13	3.35	0.14
Eating unprocessed food such as fresh meat and fruit	3.46	0.08	2.80	0.19	2.96	0.17	3.93	0.13	3.79	0.14
Eating salt	3.65	0.08	3.50	0.23	4.12	0.17	3.61	0.13	3.38	0.11
Eating fruit	3.72	0.08	3.32	0.20	3.75	0.18	3.92	0.12	3.72	0.12
Eating with other people	3.78	0.08	3.11	0.20	3.14	0.15	4.10	0.11	4.36	0.13
Concern about availability and quantity of food	3.84	0.08	3.71	0.20	3.62	0.19	3.92	0.13	4.03	0.13
Eating raw vegetables (especially leafy vegetables)	3.91	0.07	3.50	0.19	3.45	0.15	4.07	0.13	4.37	0.12
Others decide what to eat	3.94	0.05	3.84	0.14	4.26	0.12	3.83	0.09	3.83	0.10
Eating dietary fiber	4.00	0.08	4.34	0.23	4.33	0.17	3.97	0.14	3.57	0.14
Taking time preparing food	4.04	0.08	4.18	0.21	4.26	0.17	4.08	0.12	3.72	0.13
Eating meat	4.11	0.08	3.21	0.24	3.94	0.20	4.53	0.12	4.34	0.12
Eating a single meal until your stomach is full	4.45	0.08	4.86	0.20	5.12	0.15	3.82	0.13	4.30	0.13
Concern about quality of food	4.66	0.07	4.77	0.21	4.79	0.17	4.56	0.13	4.60	0.13
Considering nutritional balance while eating	4.91	0.07	5.09	0.19	4.83	0.16	4.86	0.13	4.91	0.11
Eating oils and fats	4.91	0.06	4.88	0.17	5.33	0.12	4.76	0.10	4.71	0.10
Eating an entire meal within 10 min	4.97	0.07	5.14	0.18	5.37	0.15	4.76	0.11	4.75	0.12
Eating alone	4.98	0.07	5.00	0.19	5.54	0.13	4.55	0.11	4.92	0.12
Eating a wide variety of foods	4.98	0.07	4.61	0.20	4.94	0.16	5.00	0.12	5.20	0.12
Eating meat, dairy products, and other foods that are high in saturated fats	5.01	0.07	4.96	0.19	4.81	0.15	5.18	0.11	5.02	0.12
Having trouble to decide what to eat	5.03	0.06	5.13	0.17	5.54	0.12	4.69	0.10	4.90	0.12
Each person just eats as much of what he or she wants as there are no collective rules (e.g., of the family, community, society)	5.06	0.07	5.18	0.17	5.54	0.12	4.77	0.13	4.90	0.13
Autonomy regarding eating	5.12	0.07	5.20	0.17	5.90	0.11	4.58	0.11	4.95	0.12
Being conscious of calorie content and nutritional value while eating	5.19	0.07	5.25	0.18	5.37	0.15	5.10	0.13	5.08	0.10
Most ingredients for meals are purchased	5.22	0.07	5.14	0.18	5.85	0.12	4.84	0.13	5.12	0.12
Eating high-calorie foods	5.23	0.07	5.27	0.20	6.00	0.12	4.89	0.11	4.90	0.11
Snacking frequently	5.24	0.06	5.61	0.14	5.83	0.10	4.81	0.10	4.96	0.12
Eating meat-products like ham and sausage	5.33	0.07	5.39	0.19	5.83	0.12	5.22	0.11	4.99	0.12
Consuming sugar and high-caloric sweeteners	5.43	0.07	5.84	0.16	6.21	0.09	5.05	0.10	4.93	0.13
Eating food that has been mass-produced	5.55	0.06	5.66	0.16	5.93	0.13	5.28	0.12	5.45	0.11
Eating out frequently	5.62	0.06	6.04	0.12	6.00	0.12	5.31	0.12	5.39	0.11
Eating food that has been imported from all over the world	5.64	0.06	5.95	0.12	5.96	0.12	5.37	0.12	5.48	0.12
Eating while walking	5.66	0.06	5.88	0.14	5.85	0.12	5.48	0.12	5.56	0.11
Drinking soft drinks	5.84	0.06	6.34	0.14	6.50	0.08	5.47	0.12	5.39	0.12
Being able to buy food everywhere	5.86	0.07	5.89	0.17	6.46	0.08	5.51	0.13	5.70	0.12
Eating food that is only recently eaten	5.90	0.07	5.98	0.18	6.37	0.11	5.70	0.13	5.65	0.12
Eating food that is industrially ultra-processed such as chips and ready-made meals	6.00	0.06	6.38	0.14	6.73	0.06	5.52	0.12	5.67	0.12
Frequent use of mobile phone, internet, or reading a newspaper while eating	6.33	0.06	6.73	0.09	6.89	0.04	5.89	0.13	6.07	0.12

**Table A2:** Results of a 2 × 2 MANOVA with the factors age (younger vs. older) and gender (men vs. women).

	Main Effect Age			Main Effect Gender			Interaction		
Item	*F*(1, 336)	*p*	η^2^*_p_*	*F*(1, 336)	*p*	η^2^*_p_*	*F*(1, 336)	*p*	η^2^*_p_*
Eating food that has been eaten in this area for many years ^a^	26.84	0.000	0.07	0.18	0.668	0.00	0.18	0.668	0.00
Eating grains ^a^	28.61	0.000	0.08	0.31	0.576	0.00	0.51	0.477	0.00
Strictly complying to collective rules (e.g., of the family, community, society)	13.58	0.000	0.04	0.12	0.734	0.00	0.69	0.406	0.00
Eating at home	0.39	0.533	0.00	0.59	0.443	0.00	0.07	0.793	0.00
Eating breakfast, lunch, and dinner at pre-determined times ^a^	0.02	0.886	0.00	2.04	0.154	0.01	0.08	0.780	0.00
Eating locally produced food	11.85	0.001	0.03	0.10	0.753	0.00	0.13	0.723	0.00
Eating vegetables	4.39	0.037	0.01	0.00	0.990	0.00	2.81	0.095	0.01
Having few food choices	5.22	0.023	0.02	0.03	0.861	0.00	0.21	0.646	0.00
Eating vegetables and fruit of the season	2.98	0.085	0.01	0.16	0.691	0.00	0.61	0.435	0.00
Eating unprocessed food such as fresh meat and fruit	37.68	0.000	0.10	0.00	0.948	0.00	0.89	0.345	0.00
Eating salt ^a^	4.07	0.044	0.01	1.56	0.213	0.00	7.42	0.007	0.02
Eating fruit ^a^	3.37	0.067	0.01	0.54	0.461	0.00	4.12	0.043	0.01
Eating with other people ^a^	59.56	0.000	0.15	1.07	0.302	0.00	0.61	0.434	0.00
Concern about availability and quantity of food ^a^	3.63	0.058	0.01	0.00	0.964	0.00	0.40	0.526	0.00
Eating raw vegetables (especially leafy vegetables)	25.47	0.000	0.07	0.73	0.392	0.00	1.39	0.239	0.00
Others decide what to eat	3.91	0.049	0.01	3.59	0.059	0.01	3.59	0.059	0.01
Eating dietary fiber	11.60	0.001	0.03	1.49	0.223	0.00	1.40	0.237	0.00
Taking time preparing food ^a^	4.29	0.039	0.01	0.80	0.372	0.00	2.05	0.153	0.01
Eating meat ^a^	26.51	0.000	0.07	2.59	0.108	0.01	7.56	0.006	0.02
Eating a single meal until your stomach is full ^a^	37.44	0.000	0.10	5.98	0.015	0.02	0.52	0.473	0.00
Concern about quality of food ^a^	1.62	0.204	0.00	0.04	0.852	0.00	0.01	0.943	0.00
Considering nutritional balance while eating	0.26	0.610	0.00	0.47	0.492	0.00	1.05	0.307	0.00
Eating oils and fats	9.59	0.002	0.03	2.93	0.088	0.01	4.55	0.034	0.01
Eating an entire meal within 10 min	12.90	0.000	0.04	0.60	0.439	0.00	0.72	0.398	0.00
Eating alone	15.25	0.000	0.04	11.01	0.001	0.03	0.37	0.544	0.00
Eating a wide variety of foods	4.90	0.027	0.01	3.28	0.071	0.01	0.20	0.651	0.00
Eating meat, dairy products, and other foods that are high in saturated fats ^a^	2.39	0.123	0.01	1.30	0.255	0.00	0.00	0.985	0.00
Having trouble to decide what to eat	18.28	0.000	0.05	6.14	0.014	0.02	0.64	0.423	0.00
Each person just eats as much of what he or she wants as there are no collective rules (e.g., of the family, community, society)	13.74	0.000	0.04	2.99	0.085	0.01	0.65	0.421	0.00
Autonomy regarding eating	38.13	0.000	0.10	17.96	0.000	0.05	1.77	0.185	0.01
Being conscious of calorie content and nutritional value while eating ^a^	2.57	0.110	0.01	0.13	0.718	0.00	0.26	0.612	0.00
Most ingredients for meals are purchased	14.16	0.000	0.04	12.93	0.000	0.04	2.39	0.123	0.01
Eating high-calorie foods ^a^	31.39	0.000	0.09	7.92	0.005	0.02	7.50	0.007	0.02
Snacking frequently	50.35	0.000	0.13	2.55	0.111	0.01	0.10	0.746	0.00
Eating meat-products like ham and sausage	14.74	0.000	0.04	0.63	0.427	0.00	6.41	0.012	0.02
Consuming sugar and high-caloric sweeteners ^a^	70.99	0.000	0.17	1.07	0.301	0.00	4.05	0.045	0.01
Eating food that has been mass-produced	11.10	0.001	0.03	2.88	0.090	0.01	0.14	0.705	0.00
Eating out frequently ^a^	29.88	0.000	0.08	0.03	0.856	0.00	0.22	0.636	0.00
Eating food that has been imported from all over the world ^a^	18.35	0.000	0.05	0.27	0.606	0.00	0.14	0.710	0.00
Eating while walking	7.36	0.007	0.02	0.04	0.841	0.00	0.19	0.662	0.00
Drinking soft drinks ^a^	71.44	0.000	0.18	0.12	0.731	0.00	1.06	0.305	0.00
Being able to buy food everywhere ^a^	20.09	0.000	0.06	8.85	0.003	0.03	2.22	0.137	0.01
Eating food that is only recently eaten ^a^	13.53	0.000	0.04	1.53	0.217	0.00	2.58	0.109	0.01
Eating food that is industrially ultra-processed such as chips and ready-made meals ^a^	68.34	0.000	0.17	4.70	0.031	0.01	0.76	0.385	0.00
Frequent use of mobile phone, internet, or reading a newspaper while eating ^a^	50.96	0.000	0.13	2.13	0.145	0.01	0.01	0.934	0.00

Note: ^a^ Please note that significance level of these items should be set to 0.001 as Levene’s test showed non-equal error variances for these items.
